# When Sleep and Rhythm Collide: Amiodarone, Obstructive Sleep Apnoea, and Sinus Rhythm Maintenance After Cardioversion

**DOI:** 10.31083/RCM47485

**Published:** 2026-04-08

**Authors:** Ke Wei Foong, Amr Elkammash, Daniel McKenzie

**Affiliations:** ^1^Department of Cardiology, Royal United Hospitals Bath NHS Foundation Trust, BA1 3NG Bath, UK; ^2^School of Translational Health Sciences, University of Bristol, BS8 1QU Bristol, UK

**Keywords:** amiodarone, atrial fibrillation, atrial flutter, direct current cardioversion, obstructive sleep apnoea

## Abstract

**Background:**

Recurrence of atrial fibrillation (AF) and atrial flutter (AFL) after direct current cardioversion (DCCV) remains a common problem. Several predictors of recurrence have been identified in observational studies. Current guidelines recommend considering amiodarone as an adjunct in patients at high risk of AF/AFL recurrence. However, data in the available literature on the effectiveness of amiodarone in restoring and maintaining sinus rhythm (SR) are sparse.

**Methods:**

This observational cross-sectional study analysed retrospective data from 193 patients who underwent elective DCCV for AF/AFL at a single UK cardiac centre, with follow-up at 6 weeks and 12 months. Baseline patient characteristics, including demographic data, echocardiographic findings, co-morbidities, and SR maintenance rate, were compared between patients treated with amiodarone and those without. Multivariate logistic regression was performed to identify parameters associated with DCCV failure.

**Results:**

A total of 13.0% of the study population were started on amiodarone before DCCV. Those on amiodarone were more likely to have had a previous failed DCCV (60.0% vs. 21.4%; *p *< 0.001), AF/AFL duration of at least 12 months (84.0% vs. 53.0%; *p *= 0.003), a left ventricular ejection fraction (LVEF) of less than 40% (32.0% vs. 14.3%; *p *= 0.03), and a diagnosis of coronary artery disease (CAD) (32.0% vs. 13.7%; *p *= 0.02). Treatment with amiodarone was not associated with an increased rate of SR restoration at the time of DCCV (96.0% vs. 92.3%; *p *= 0.50). However, amiodarone treatment was associated with SR maintenance at 6 weeks (92.0% vs. 54.8%; *p *< 0.001) and at 12 months (60.0% vs. 26.8%; *p *< 0.001). Multivariate logistic regression analysis identified obstructive sleep apnoea (OSA) as the only parameter associated with DCCV failure (adjusted odds ratio (OR) 10.5; 95% confidence interval (CI) 2.5–53.5; *p *= 0.005). There was an increased risk of peri-procedural bradyarrhythmia with amiodarone therapy (adjusted OR 8.85; 95% CI 1.84–42.7; *p* = 0.007).

**Conclusions:**

Amiodarone treatment is associated with maintenance of SR following elective DCCV for AF/AFL. This effect is observed even in patients with risk factors for recurrence, including previous failed DCCV, longer AF/AFL duration, and reduced LVEF. OSA is an independent predictor of DCCV failure; further research is required to delineate the role of early adjunctive amiodarone therapy in these patients.

## 1. Introduction

Rhythm control remains a vital strategy in the management of atrial fibrillation (AF) and atrial flutter (AFL), aiming to relieve symptoms and prevent adverse cardiovascular outcomes. Elective direct current cardioversion (DCCV) is a commonly used procedure to restore sinus rhythm (SR) in patients with persistent AF/AFL, with the aim of alleviating symptoms related to AF/AFL without a mortality benefit [1]. However, maintaining SR post-DCCV is challenging due to atrial remodelling, electrical heterogeneity and associated clinical variables that trigger arrhythmia [2]. These factors may jeopardise the long-term maintenance of SR despite the success of acute cardioversion [2].

Post-DCCV antiarrhythmic therapy can influence the effectiveness of this rhythm control strategy. Several randomised controlled trials and meta-analyses have found that amiodarone is more effective in maintaining SR after DCCV than other antiarrhythmic medications or no therapy [3]. On the other hand, use of amiodarone was associated with increased incidence of slow heart rates and other adverse events necessitating careful patient selection and monitoring [4, 5].

The presence of associated co-morbidities, such as obstructive sleep apnoea (OSA), also affects the success of DCCV. OSA causes atrial structural and electrical remodelling through intermittent hypoxia, sympathetic overstimulation, and inflammation, leading to persistence of AF/AFL and increased incidence of DCCV failure [6]. Other cardiovascular risk factors, such as AF/AFL duration, obesity, and heart failure, further undermine the success of DCCV [7].

Concerns about the adverse effects related to amiodarone treatment and the impact of associated medical co-morbidities on the success of DCCV have inspired us to design a cross-sectional study to understand the relationship between amiodarone treatment, the success of acute cardioversion, and the maintenance of SR. We also studied the impact of various medical co-morbidities on immediate DCCV success and the number of shocks required to restore SR. Understanding these relationships can enrich the limited literature on the topic and inform tailored treatment strategies to optimise rhythm control and minimise complications associated with DCCV.

## 2. Study Summary

This observational cross-sectional study was performed at Royal United Hospital, Bath, UK, and enrolled 193 consecutive patients admitted for elective DCCV for AF/AFL between November 2020 and March 2023. If a patient underwent DCCV more than once during this time period, they were included only once and data from the latest procedure was used. Patients presenting with arrhythmias other than AF/AFL, those who failed to provide informed consent for using their data for research purposes, or those who experienced spontaneous restoration of SR prior to DCCV were excluded from analysis. The study was carried out in accordance with the guidelines of the Declaration of Helsinki and approved by the Ethics Committee of Royal United Hospital Bath NHS Foundation Trust (Protocol No. CARD/QI/2025-26/02). As this article is a retrospective study, patient informed consent statement was waived.

### Statistical Analysis

Power analysis of the sample size showed that 139 subjects were needed to provide results with a margin of error of 5% and a confidence interval (CI) of 95% according to the reported DCCV failure rate of 10% in the available literature [8]. Clinical data were collected using a standardised electronic proforma including demographic data, current antiarrhythmic therapy, left ventricular ejection fraction (LVEF), medical co-morbidities, body mass index (BMI), N-terminal pro-B-type natriuretic peptide (NT-proBNP) levels, and left atrial (LA) volumes. Where more than one result was available for any parameter, the most recent result before DCCV was recorded. For arrhythmia assessment, the duration of AF/AFL was recorded in each case, along with the outcomes from DCCV, namely immediate success, number of shocks required to restore SR, and maintenance of SR at 6 weeks and 12 months after the index procedure (Table 1).

**Table 1.** **Baseline patient characteristics before DCCV**.

Variable		Value
Demographics		
	Age (years), mean (±SD)	66.4 (±9.8)
	Male, n (%)	148 (76.7)
	BMI (kg/m^2^), mean (±SD)	30.2 (±5.9)
Diagnoses and medical co-morbidities		
	Atrial fibrillation, n (%)	161 (83.4)
	Atrial flutter, n (%)	29 (15.0)
	Diabetes mellitus, n (%)	29 (15.0)
	Hypertension, n (%)	95 (49.2)
	Thyroid disease, n (%)	14 (7.3)
	COPD, n (%)	10 (5.2)
	Valvular disease, n (%)	51 (26.4)
	OSA, n (%)	14 (7.3)
	CAD, n (%)	31 (16.1)
	Congenital heart disease, n (%)	6 (3.1)
	Cardiomyopathy, n (%)	14 (7.3)
Laboratory and echocardiographic findings		
	NT-proBNP (ng/L), mean	2600.53
	LVEF (abs %), mean	50.77
	Average E/e′, mean	9.58
	LA volume (mL), mean	87.72
Medications		
	Amiodarone, n (%)	25 (13.0)
	Digoxin, n (%)	53 (27.5)
	Flecainide, n (%)	17 (8.8)
	Beta-blocker, n (%)	165 (85.5)
	Calcium channel blocker, n (%)	24 (12.4)
Outcomes		
	Successful restoration of SR, n (%)	179 (92.7)
	≥2 shocks required, n (%)	69 (35.8)
	Peri-procedural bradyarrhythmia, n (%)	7 (3.6)
	Maintained SR at 6 weeks, n (%)	115 (59.6)
	Maintained SR at 12 months, n (%)	60 (31.1)

BMI, body mass index; CAD, coronary artery disease; COPD, chronic obstructive pulmonary disease; LA, left atrium; LVEF, left ventricular ejection fraction; NT-proBNP, N-terminal pro-B-type natriuretic peptide; OSA, obstructive sleep apnoea; SD, standard deviation; SR, sinus rhythm.

Continuous variables are presented as mean ± standard deviation or median (interquartile range), as appropriate, and categorical variables as counts and percentages. Between-group comparisons were performed using the independent samples *t*-test or Mann–Whitney U test for continuous variables and the chi-square or Fisher’s exact test for categorical variables. Multivariable logistic regression models were constructed to identify independent predictors of failed DCCV and peri-procedural bradyarrhythmia. Candidate variables included those with established clinical relevance (age, sex, AF/AFL duration, prior failed DCCV, LVEF, coronary artery disease (CAD), OSA) and those associated with the outcome on univariable analysis at *p* < 0.10. Results are reported as odds ratios (OR) with 95% CI. Analyses were performed using a complete-case approach as the proportion of missing data for individual covariates was low; no imputation was undertaken. Given the modest sample size and the exploratory nature of secondary comparisons, no formal adjustment for multiple testing (e.g., Bonferroni correction) was applied. Owing to the relatively small number of patients treated with amiodarone, propensity-based methods such as matching or inverse probability weighting were not applied to avoid substantial loss of observations and unstable estimates. We used a multivariable logistic regression model, including clinically relevant covariates, to adjust for baseline imbalances. We analysed the data using IBM SPSS 28.0 software (IBM, Chicago, IL, USA).

## 3. Study Results

**Fig. 1.**
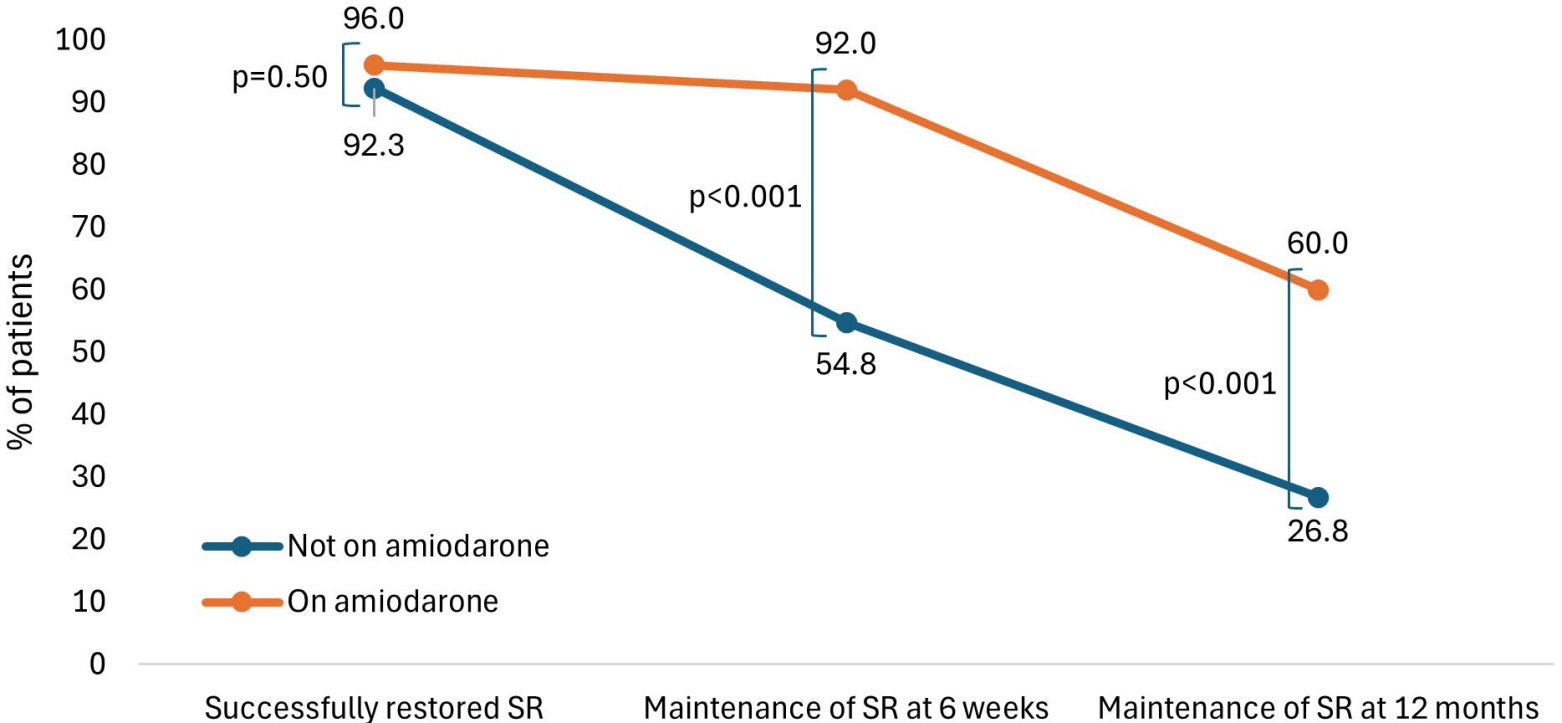
**Percentage of patients in SR immediately after cardioversion, at 6 weeks, and at 12 months, with and without amiodarone treatment**.

Patients on amiodarone were more likely to have had at least one previous failed DCCV (60.0% vs. 21.4%, *p* < 0.001), AF/AFL duration of 12 months or more (84.0% vs. 53.0%, *p* = 0.003), LVEF of less than 40% (32.0% vs. 14.3%, *p* = 0.03), and CAD (32.0% vs. 13.7%, *p* = 0.02). Treatment with amiodarone was not associated with an increased rate of SR restoration at the time of DCCV (96.0% vs. 92.3%, *p* = 0.50). However, there was an association between treatment with amiodarone and SR maintenance at 6 weeks (92.0% vs. 54.8%, *p* < 0.001) and at 12 months (60.0% vs. 26.8%, *p* < 0.001) (Fig. 1).

**Fig. 2.**
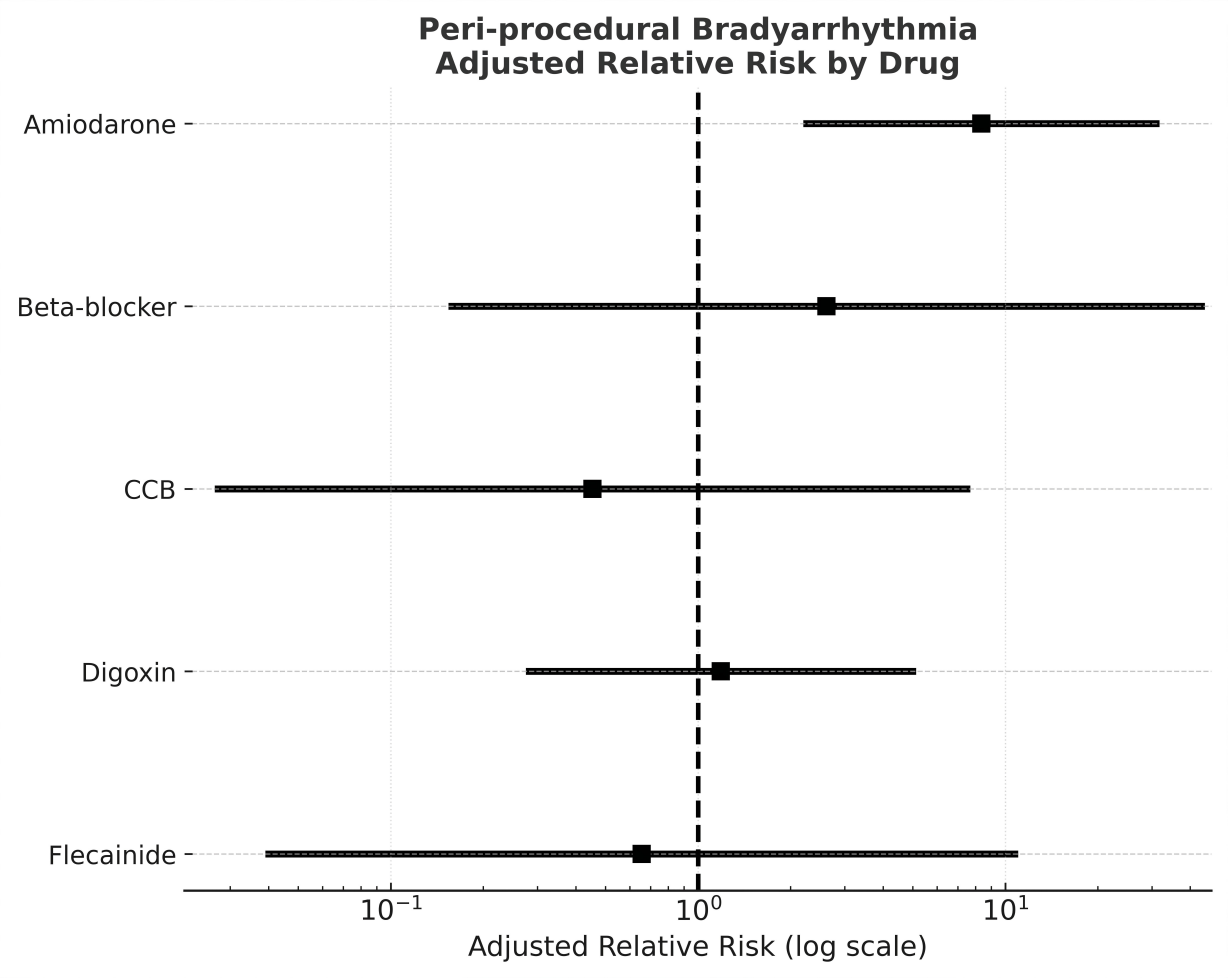
**Forest plot showing the adjusted relative risk (RR) of peri-procedural bradyarrhythmia associated with different anti-arrhythmic drugs**.

Patients on amiodarone were more likely to develop peri-procedural bradyarrhythmia than those not on amiodarone, (16.0% vs. 3.0%, *p* = 0.02). This finding was not found with other antiarrhythmic drugs (beta-blockers, calcium channel blockers, flecainide, and digoxin) (Fig. 2). Analysis by multivariate logistic regression, adjusted for potential confounders, showed that amiodarone treatment was independently associated with increased risk of peri-procedural bradyarrhythmia (adjusted OR 8.85; 95% CI 1.84–42.7, *p* = 0.007).

Importantly, we found that OSA was the only parameter among those studied associated with increased risk of failure of DCCV (Table 2). The logistic regression analysis confirmed that it was an independent predictor of failed DCCV (adjusted OR 10.5; 95% CI 2.5–53.5, *p* = 0.005). We also identified a positive correlation between OSA and number of shocks needed to restore SR during the DCCV session (Point biserial correlation coefficient, r = 0.2, *p* = 0.006).

**Table 2.** **Association between different clinical parameters and failure of DCCV**.

Parameters	Adjusted OR	95% CI	*p*-value
Hypertension	1.84	0.58–5.82	0.512
Diabetes	0.04	0.002–1.00	0.1
Thyroid disease	0.54	0.03–9.34	0.28
COPD	3.14	0.47–20.95	0.73
Valvular disease	0.34	0.07–1.71	0.9
CAD	0.51	0.09–2.93	0.35
OSA	10.5	2.5–53.5	0.005
LA dilatation	0.60	0.18–1.99	0.96
LVEF <40%	3.19	0.78–13.10	0.61

## 4. Discussion

Our study found that adjunctive therapy with amiodarone is associated with effective maintenance of SR even in patients with one or more risk factors for recurrence of AF/AFL, including previous failed DCCV, longer AF/AFL duration, and reduced LVEF. In addition, we identified OSA as an independent risk factor for DCCV failure, highlighting a patient group that warrants greater consideration of the need for adjunctive antiarrhythmic therapy. Our study showed an increased incidence of peri-procedural bradyarrhythmia associated with amiodarone, although most of these resolved spontaneously without specific treatment.

The optimum dosing and duration of amiodarone therapy after successful DCCV remains a subject of investigation [9, 10]. Given that amiodarone-related side effects are typically more common with higher doses and longer duration of administration [10], further research is needed to determine whether a minimum duration of amiodarone therapy post-DCCV is required to optimise long-term maintenance of SR while minimising the risk of side effects.

The association between OSA and the development of AF/AFL has been well described, and existing observational studies also suggest that chronic intermittent hypoxia resulting from OSA leads to electrical and structural atrial remodelling, thereby reducing the efficacy of rhythm control treatments [11]. Due to the relatively small number of patients with OSA in our study, it was not feasible to analyse in greater detail the impact of amiodarone on the maintenance of SR in these patients. Such patients may benefit from early referral for sleep studies and OSA treatment before pursuing rhythm control treatments [12]. In addition, given that the effectiveness of continuous positive airway pressure (CPAP) treatment is limited by poor compliance, adjunctive antiarrhythmic therapy with amiodarone may play a larger role in this patient group, including during the first attempt at DCCV.

## 5. Limitation

Despite the clinical value of the study’s results, it had several limitations. First, the patients were not randomised to amiodarone treatment. Treatment was based on clinical assessment, which may have confounded the results despite multivariable adjustment. Second, the study had some statistical limitations. The study was not powered to examine the interactions between OSA, amiodarone therapy, and DCCV outcomes. The small and unbalanced study cohort made propensity score matching or weighting unfeasible. We did not perform formal corrections for multiple testing of variables, so all secondary analyses should be interpreted as exploratory and hypothesis-generating, given the increased risk of type I error. The detailed time-to-event data for AF/AFL recurrence were unavailable, so we could not perform Cox proportional hazards analyses to examine differences in the timing of AF/AFL recurrence. Instead, our models were based on binary rhythm status at fixed follow-up time points. Third, details on OSA treatment status or CPAP adherence were not systematically collected. The absence of this data prevented the study of differences in outcomes between the treated and untreated OSA subgroups. It also did not allow for the assessment of differences in amiodarone effectiveness between subgroups. Fourth, all patients were recruited from a single UK cardiac centre, which jeopardised the generalisability of the study’s findings to other clinical settings.

## 6. Conclusions

Adjunctive therapy with amiodarone before and after elective DCCV for AF/AFL was associated with higher rates of SR maintenance in this observational study. This advantage included patients with risk factors for recurrence, such as previous failed DCCV, longer AF/AFL duration and reduced LVEF.

There is an increased risk of self-limiting peri-procedural bradyarrhythmia with amiodarone. OSA is associated with failure of DCCV for AF/AFL and an increased number of shocks needed to restore SR. Further prospective randomised studies are required to delineate the optimum timing, dosing, and duration of amiodarone therapy in these patients, and to study the impact of OSA treatment on DCCV outcomes.

## Data Availability

All data reported in this paper will be shared by the lead contact upon request.

## Abbreviations

AF, atrial fibrillation; AFL, atrial flutter; BMI, body mass index; CAD, coronary artery disease; CI, confidence interval; COPD, chronic obstructive pulmonary disease; CPAP, continuous positive airway pressure; DCCV, direct current cardioversion; ECG, electrocardiogram; LA, left atrium; LVEF, left ventricular ejection fraction; NT pro-BNP, N-terminal pro-B-type natriuretic peptide; OR, odds ratio; OSA, obstructive sleep apnoea; RR, relative risk; SR, sinus rhythm.
